# The prevalence and determinants of HIV seroconversion among booked ante natal clients in the University of Uyo teaching hospital, Uyo Akwa Ibom State, Nigeria

**DOI:** 10.11604/pamj.2016.25.247.6715

**Published:** 2016-12-21

**Authors:** Nsikak Paul Nyoyoko, Augustine Vincent Umoh

**Affiliations:** 1 Department of Obstetrics and Gynaecology, University of Uyo Teaching Hospital, Uyo, Nigeria

**Keywords:** HIV, seroconversion, determinants, ante natal care, HCT, PMTCT, prevalence, window period, Uyo, Nigeria

## Abstract

**Introduction:**

The Prevention of Mother to Child transmission (PMTCT) of HIV, an important intervention in the fight against HIV/AIDS can only be of benefit if the HIV status of the mother is known. Unfortunately, some women receive HIV counseling and testing (HCT) during the window period or prior to new infection in pregnancy and therefore, miss-out on the gains of PMTCT. A repeat HIV test would identify this later seroconversion and ensure early intervention. This study aimed at determining the prevalence of HIV seroconversion during pregnancy and assessing the factors associated with HIV seroconversion in clients who attended ante-natal clinic (ANC) in University of Uyo Teaching Hospital, Uyo.

**Methods:**

A descriptive cross-sectional design was used to study 502 HIV negative clients who consecutively presented for ANC in the University of Uyo Teaching Hospital Uyo over a 3 months period (July -September 2013).

**Results:**

Fifteen (3%) of the 502 women who were HIV negative at booking visit tested positive (seroconverted) to HIV. Determinants of seroconversion at multivariate level were age bracket of 25-29 years (OR: 3.20; 95%CI: 2.39-4.29) clients’ partners who had sexually transmitted infections, as evidenced by penile discharge (OR: 0.09; 95%CI: 0.01-0.45); Clients in polygamous setting (OR: 3.98; CI: 1.64-9.65); those taking alcohol (OR: 0.12; CI: 0.20-0.80) and those with partners taking recreational drugs (OR: 0.05, 95%CI: 0.002-0.995).

**Conclusion:**

The study revealed a high seroconversion rate. Re-screening of pregnant women after the booking HCT is a very cost effective and beneficial strategy for the elimination of mother to child transmission of HIV.

## Introduction

The human immunodeficiency virus (HIV) pandemic is one of the most serious public health crises the world faces today. Acquired immunodeficiency syndrome (AIDS) has killed more than 25 million people since it was first discovered in 1981 [1]. In 2008, approximately 33.4 million people worldwide (1% of the global adult population aged 15-49 years) were infected with HIV with 67% of all people living with HIV worldwide residing in sub-Saharan Africa and 91% of all new infections among children occur there [2]. HIV/AIDS remains a threat to health in Nigeria and continues to strain the struggling health system as it has reversed many developmental gains of the recent past including maternal and under-five mortality rates. With an estimated population of 162,265,000 as at mid-2011 and an annual growth rate estimated to be 2.6% in 2012 [3], Nigeria, the most populated country in sub-Saharan Africa has the second largest number of people living with HIV in the world [4] with a national HIV prevalence of 4.1% and 3.4% in 2010 and 2012 respectively [5]. Akwa Ibom state HIV prevalence in both surveys were 10.9% and 6.5% respectively with a corresponding national position of second and sixth respectively [5]. It is pertinent to note however, that study of 2010 was a hospital based sentinel study while the 2012 study was a population based study. The prevention of mother-to-child transmission (PMTCT) of HIV focuses mainly on women who are HIV-positive at their first antenatal visit with uncertainty about the contribution of mothers who undergo sero-conversion after their first antenatal visit and during breastfeeding to the overall vertical transmission rate. Acute maternal HIV-1 infection during pregnancy and breast-feeding is associated with a very high rate of mother-to-child transmission (MTCT) of HIV-1. This rate can be over 50% in breast-feeding populations; approximately one third of infants whose mothers seroconvert following delivery will become infected through breast-feeding alone [6, 7]. Such a high transmission rate is understandable because maternal viral load is the most consistent predictor of MTCT of HIV infection [8], and acute HIV infection is associated with very high levels of HIV-1 RNA [9]. Most of this sero-conversion is unrecognized and therefore untreated. Despite recommendations from the (Nigerian) National Guidelines on PMTCT [10], repeat HIV testing during pregnancy is performed infrequently for women who have negative ante partum HIV test results. Women who sero-convert to HIV-positive status during pregnancy do not therefore receive prophylaxis because they are erroneously believed to be HIV-negative. Especially at risk are women who book early in pregnancy, increasing the interval between testing at booking visit and delivery. These women would also not receive counseling on the best method of infant feeding, thus increasing the risk of transmission to their infants. The risk of transmission of HIV to health workers attending to these women is also increased as they may still be treated as HIV negative. Repeat HIV testing during late pregnancy or labour could identify these cases and thus is very necessary. Defining co-factors for HIV infection during pregnancy and the postpartum period is a useful first step towards development of strategies to decrease HIV acquisition to both mothers and infants during this period [11]. Guidelines in resource-limited settings like ours recommend HIV testing as early as possible during pregnancy and then repeat testing toward the end of pregnancy (at about 36 weeks' gestation) or during labour; a strategy that may be cost-effective [12]. In their analysis of the cost-effectiveness of repeat HIV testing in pregnancy, Samson and colleagues [13] recommended that HIV testing be repeated during the third trimester in settings where HIV-1 incidence is 1.2 per 1000 person-years or higher. At that incidence, the costs of a second test are offset by averted medical costs. This study sought to determine the prevalence of HIV sero-conversion among women attending ANC in University of Uyo Teaching Hospital, Uyo and establish the factors associated with the sero-conversion. It is envisaged that the findings of this study would help capture mothers newly infected with HIV and recommend them for HIV care during pregnancy as well as decrease MTCT of HIV during pregnancy.

## Methods

**Study location:** The Study was conducted in the maternity complex of the University of Uyo Teaching Hospital, Uyo (UUTH). This Hospital is the only tertiary healthcare facility in Akwa Ibom State, South-South geopolitical zone of Nigeria providing specialized health care services to the over 4 million people of the state and its environs including PMTCT intervention to HIV positive pregnant women. In the year 2011, there were 3,736 antenatal clients and 2,349 deliveries in the facility, with 604 deliveries in the 3 months of the study – July to September 2011.

**Study population and technique:** HIV sero-negative clients who booked for ANC at a gestational age earlier than 26 weeks gestation were consecutively recruited over a 12 weeks period until the sample size was obtained.

**Data collection and research instrument:** A semi-structured interviewer-administered questionnaire was used to obtain the bio-data of clients and that of their partners/spouses; as well as the risk factors for HIV transmission. HIV Re-testing was done for the selected clients in labour, before induction of labour or prior to an elective caesarean section; according to the national HIV Rapid Testing Serial Algorithm.

**Data analysis:** Data obtained was analyzed using the statistical package for Social Sciences (SPSS) version 17. The level of significance was set at p-value less than 0.05.

**Ethical consideration**: Ethical approval was obtained from the ethical committee of UUTH, Uyo.

## Results

**Figure 1:**
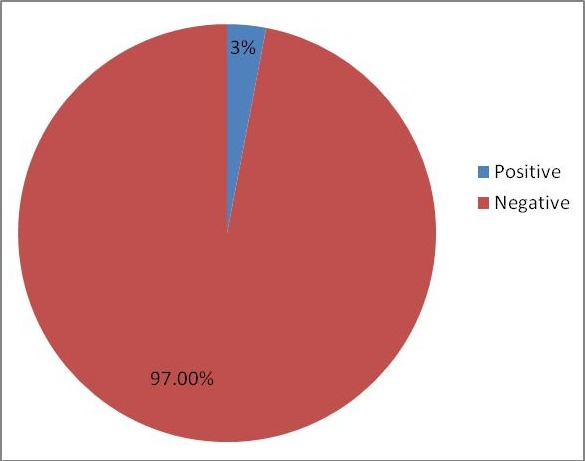
Prevalence of HIV seroconversion among booked antenatal clients at delivery in university of Uyo teaching hospital, Uyo, 2013

The total number of booked clients at delivery for the 3 months when the study was conducted was 604. Thirty six (36) of the 604 (6%) were known HIV positive clients, and were exempted from the study. Five hundred and two HIV negative clients were recruited into the study. Of these, 15 (3%) tested positive (seroconverted) when re-tested for HIV infection in labour as shown in Figure 1. The Majority of respondents were in the age bracket 25-29 years with a mean age of 29.46 + 3.99 years. Among those who seroconverted, majority (n=6; 40%) were within the 25-29 year age bracket. Most (n=440; 87.6%) respondents resided in urban areas. Four hundred and ninety two (98%) clients were married. Every client (100%) had a form of formal education with a higher proportion (n = 372; 74.1%) having tertiary (post-secondary) education. Almost an equal proportion were professionals (n=189; 37.6%) and unemployed (n=187; 37.3%). Majority (n=299; 59.6%) were Ibibios. Primigravid clients accounted for 43.4 % (n=218) of women recruited while only 2.8 % (n=13) were grandmultiparous. The knowledge of respondents concerning HIV transmission is displayed in Table 1. Two (0.4%) respondents were unaware of sexual transmission of HIV, 3 (0.6%) did not know it could be transmitted via blood transfusion and 25 (5%) were unaware of mother to child transmission of HIV infection. Risk factors for HIV sero-conversion analyzed is shown on Table 2. 16 (3.2%) clients had blood transfusion in index pregnancy. Thirteen (2.6%) had unprotected sexual intercourse with people other than their partners. Of the 153 (30.5%) clients who take alcohol, 26 (17%) had been drunk. Only 3 (0.6%) women smoke and none use recreational drugs. Relating sero-conversion to risk factors (as shown in Table 3) found in clients who became HIV positive at re-testing, there was no statistically significant relationship between HIV status at delivery and lack of condom use in pregnancy (p =0.255), smoking habit(p =0.760) and partner’s alcohol intake (p= 0.423). However, unprotected intercourse with persons other than their partners (p =0.008), having partners with multiple sexual partners (0.001), alcohol intake (p = 0.002), and those who lived separate from their husbands (p = 0.001) were significantly at risk of HIV sero-conversion in pregnancy. Other statistically significant association includes partner with penile discharge, knowledge of partner’s HIV status, partner’s smoking habit, recreational drug use and, number of wives (p = 0.001) (Table 4). At multivariate analyses however, few factors found to be statistically significant in their association with HIV seroconversion were: clients of age group 25-29 years (OR: 3.20; 95%CI: 2.39-4.29; p < 0.001), client’s alcohol intake (p=0.03; OR=0.108), number of partner’s wives (p=0.02; OR=0.02), partner use of recreational drugs (p=0.05; OR =0.05) and penile discharge in partner (p=0.002; OR=0.036) (Table 5).

**Table 1:** Knowledge of antenatal clients in UUTH on the modes of transmission of HIV/AIDS (n=502)

Variables	Frequency n (%)	
	Yes n (%)	No n (%)
HIV can be sexually transmitted	500 (99.6)	2(0.4)
HIV can be transmitted through Blood transfusion	499(99.4)	3(0.6)
HIV can be transmitted from mother to child	477(95.0)	25(5.0)

**Table 2:** Risk factors for HIV among antenatal clients in UUTH, 2013 (n=502)

Risk Factors	Yes n (%)	No n (%)
Received blood transfusion in index pregnancy	16(3.2)	486(96.8)
Unprotected sexual intercourse with other Partners in index pregnancy	13(2.6)	489(97.4)
Take alcohol in index pregnancy	153(30.5)	349(69.5)
Clients Ever been drunk in index pregnancy (n=153)	10(6.5)	143(93.5)
Smoking in index pregnancy	3(0.6)	499(99.4)
Take recreational drugs in index pregnancy	0(0)	502(100)

**Table 3:** Relationship between antenatal clients HIV status at delivery and risk factors for seroconversion - client factors (n =502)

Variable	HIV positive n (%)	HIV Negative n (%)	Statistical test and p values
**Condom use in index pregnancy**			
Always	0(0)	2(100)	X2=4.06
Never	11(2.5)	429(97.5)	P=0.255
No intercourse	0(0)	5(100)	
Sometimes	4(7.3)	51(97.7)	
**Clients with other sex partners**			
No other sex partner	13(2.7)	476(97.3)	X2=7.075
Have unprotected sexual intercourse	2(15.4)	11(84.6)	P=0.008
**Respondents alcohol status**			
Don’t take alcohol	5(1.4)	344(98.6)	X2=9.557
Take alcohol	10(6.5)	143(93.5)	P=0.002*
**Ever been drunk (n=153)**			
Yes	2(11.1)	16(88.9)	X2=0.699
No	8(5.9)	127(94.1)	P=0.403
**Respondents smoking status**			
No	15 (3.0)	484(97.0)	X2=0.093
Yes	0 (0.0)	3 (100.0)	P=0.760
**Blood transfusion in pregnancy**			
Not transfused blood	15 (3.1)	471(96.9)	X2=0.509
Transfused blood	0(0)	16 (100)	P=0.476

**Table 4:** Relationship between antenatal clients HIV status at delivery and risk factors for sero conversion – partner factors (n =502)

**Partners have other sexual partners**			
Don’t know	7(5.9)	112 (94.1)	X2=21.759
No	6 (1.6)	370 (98.4)	P=0.00^+^
Yes	2 (28.6)	5 (71.4)	
**Respondents living together with partner**			
No	4(13.8)	25(86.2)	X2=12.396
Yes	11(2.3)	462 (97.7)	P=0.00^+^
**Partner with penile discharge**			
Don’t know	7(17.9)	32 (82.1)	X2=32.733
No	8 (1.8)	447 (98.2)	P=0.00^+^
Yes	0 (0)	8 (100.0)	
**Partner s who know their HIV Status**			
Don’t know	2(2.9)	68 (97.1)	X2=27.543
No	8 (14.0)	49 (86.0)	P=0.00^+^
Yes	5 (1.3)	370 (98.7)	
**Partners true HIV status**			
Positive	1 (100)	0 (0)	X2=74.198
Negative	4 (1.1)	370 (98.9)	P=0.013^+^
**Partner take alcohol**			
No	6(2.4)	245(97.6)	X2=0.618
Yes	9(3.6)	242(96.4)	P=0.432
**Partners ever been drunk (n=251)**			
No	5 (2.4)	202 (98.6)	X2=4.677
Yes	4 (9.1)	40(90.9)	P=0.53
**Partners smoking status**			
Don’t smoke	11 (2.3)	460 (97.7)	X2=11.206
Smoke	4 (12.9)	27 (87.1)	P=0.001^+^
**Partner use of recreational drugs**			
Don’t use	13 (2.6)	478 (97.4)	X2=8.956
Use	2 (18.2)	9 (81.8)	P=0.003*
**No. of partner’s wives**			
1	12(2.4)	484(97.6)	X2=72.173
2	3(75.0)	1 (25.0)	P=0.00^+^
3	0(0)	2 (100)	

**Table 5:** Univariate and multivariate analysis of variables that predicts HIV sero- conversion of HIV status of antenatal clients at delivery in UUTH, Uyo, 2013

Variable	*Univariate Analysis*			Multivariate Analysis		
	*OR*	*95%CI*	*Pvalue*	OR	95%CI	Pvalue
Live together with partner	6.72	1.99-22.60	*0.002*	3.70	0.60-22.87	0.159
Penile discharge	0.28	0.17-0.49	*0.000*	0.09	0.01-0.45	0.004^+^
Other sex partners	*0.51*	*0.30-0.86*	*0.012*	0.23	0.01-6.22	0.389
Partner smokes	*0.16*	*0.04-0.54*	*0.003*	1.34	0.08-22.09	0.836
Partner use recreational drug	*0.12*	*0.02-0.62*	*0.011*	0.05	0.002-0.995	0.05^+^
client has unprotected sex with others	*0.15*	*0.03-0.75*	*0.021*	3.83	0.13-112.19	0.436
Partner with many wives	*0.01*	*0.00-0.86*	*0.000*	3.98	1.64-9.65	0.000^+^
Condom use in index pregnancy	*0.32*	*0.10-1.06*	*0.06*	0.25	0.03-1.73	0.162
Have vulva vaginal ulcer	*0.08*	*0.01-0.44*	*0.004*	0.09	0.008-1.22	0.071
Age group (25-29yr)	*2.19*	*6.09-7.89*	*0.00*	3.20	2.39-4.29	0.000^+^
Clients who take alcohol	*0.21*	*0.07-0.62*	*0.005*	0.14	0.25-0.80	0.027^+^

+ = significant

## Discussion

The recommendation of a double HIV antibody test algorithm [12, 14] (one in early pregnancy and a repeat in late pregnancy) to reduce missed opportunities for diagnosis and PMTCT interventions, may not be out of place for a country like Nigeria. This study was designed with this in view. This study documents a repeat HIV sero-prevalence of 3.0% among the booked antenatal attendees who were previously HIV sero-negative earlier in pregnancy. This figure conforms to the CDC's cut off of > 1.2% for high risk populations [14].This prevalence was higher than that in Jos University Teaching Hospital, Jos (JUTH) with a seroconversion rate of 2.1% (5 out of 235 women) [15]. The sample size of the Jos study, being smaller, may have contributed to this difference especially considering the fact that there are at least 3 referral health centres located in Jos metropolis catering for women in and around Jos. This is unlike in Uyo where UUTH is the only tertiary referral Centre. Also, Uyo (Akwa Ibom state) had a higher HIV prevalence than Jos (Plateau State) in the National HIV sentinel survey report 2012 [5] and expectedly, should have a higher HIV seroconversion rate in pregnancy. A recent study [16] closer to Uyo, in Nnewi –south east Nigeria revealed a repeat HIV sero-prevalence of 3.91% which is close to the result obtained here. It is important to note the sociocultural similarities between these 2 regions and the fact that both studies were carried out in tertiary health facilities may be responsible for the similar sero-conversion rate. In Eastern and Southern African countries, the seroconversion rates were varied with retest prevalence of 2.9% in Botswana [17], 2.6% in Kenya [11], 4.4% in Swaziland [18] and 3.0% in South Africa [19]. These studies produced results similar to this study, though they were multi-centered and carried out in both urban and rural areas. Also, the women were followed-up and re-tested at various times postpartum, 12 to 60 weeks after initial test. The length of follow-up affects the cumulative prevalence rate because the window period varies in individuals. For instance, a Zimbabwean study [20] where repeat tests were conducted at delivery, six weeks postpartum and at three monthly intervals until 24 months or on termination due to subsequent pregnancy, death or loss to follow up; gave a seroconversion rate as high as 17.7% (4.8 per 100 person years; 95% confidence interval [CI], 3.1 to 6.5).

The high HIV prevalence in some of the study areas affected the seroconversion rate. For instance, women in Western Kenya [21], where the HIV prevalence rate of 15% is over twice the national rate, were at especially high risk for seroconversion during pregnancy. In settings with high HIV incidence, new maternal infections may actually be responsible for a substantial proportion of HIV seroconversion and MTCT of HIV, particularly when MTCT prevention programs are effectively reducing transmission among mothers with known HIV infection. These women represent the missed opportunities for PMTCT of HIV. For example, it has been estimated that MTCT secondary to seroconversion during pregnancy could account for more than 40% of all ongoing MTCT in Botswana [17]. Investigators who conducted these studies above, acknowledge that some of the women who first tested positive late in pregnancy or in the early postpartum period may have had an initial false-negative result (or may have been in the “window period” for seroconversion). Nevertheless, a second true-positive test represents an important (albeit late) opportunity to intervene to prevent MTCT of HIV infection. In this study, the risk factors to HIV transmission common to the study area were analyzed. As already known, pregnancy in itself is a risk factor especially when conceived through heterosexual intercourse.

In the 2010 HIV survey in Nigeria [5], the infection was more prevalent among the 20-39 year age group and more specifically, mostly found in the 30-34 year age group. In the Uyo and a Tanzanian study [22], the 25-29 year age group was most affected. The Nnewi study [16] also showed a modal age group of 25-29 years. However, in Harare, Zimbabwe [20] women aged 17 years and below had the highest seroconversion incidence (6.25%) followed by those aged 18 to 19 years (5.42%). This suggests that most infections in women occur at a younger age, during the first few years after sexual debut. Biological factors such as immature genital tract and cervical ectopy which is common in young women might increase the risk. In 2010, HIV prevalence was highest in urban areas of Nigeria [5]. This study in UUTH conforms to the national findings, but this could simply be attributed to the fact that the study area, UUTH Uyo, is urban. In a study in KwaZulu-Natal, South Africa [19], among the 2377 HIV-negative women retested, 1099 (46.2%) and 1278 (53.4%) were tested at urban and rural health facilities, respectively; HIV incidence in pregnancy was higher but not statistically significant at the urban facilities (12.4/100 woman years versus 9.1/100 woman years). Univariate analysis showed an association between HIV seroconversion and unprotected sexual intercourse with multiple sexual partners (OR: 0.51, 95%CI: 0.3-0.86; p=0.012). This was similar to a study in northern Nigeria [23] (AOR 2.4, 95% CI= 0.97- 6.2) and Tanzania [22] (p < 0.001). However using multivariate analysis, this association was no more significant. Same pattern was noted when association was sought for client living with partner. In a South African study [24], lack of co-habitation was one of the five major factors associated with HIV seroconversion in pregnancy in over 80% of cases. Lack of co-habitation increases the risk of having multiple sexual partners, thus creating an avenue for HIV transmission. Smoking and vulval/vaginal ulcers were predictive of HIV seroconversion by univariate analysis, but not so with multivariate. This agrees with a study in Jos [23], Nigeria stating that HIV infection was not found to be associated with smoking in women and history of sexually transmitted infection.

In this study, there was a strong association between HIV seroconversion with clients who drink alcohol but none for partners who drink alcohol. This is contrary to the findings in Jos [23] where no association was found between HIV seroconversion and alcohol use in patients and their partner. In Uganda [25] however, individuals who had at any point, consumed alcohol experienced HIV prevalence twice that of those who had never done so: 10% vs 5% (OR 2.0, 95% CI: 1.5-2.8). Alcohol abuse has been reported to be associated with having a greater number of sex partners, history of condom failures, and lifetime history of sexually transmitted infections [26]. Use or non-use of condoms was not found to be associated with HIV seroconversion in this study. Contrary to this finding, in Harare [20], women who did not seroconvert during the time of pregnancy or follow up, were significantly more likely to have used a condom with their partners (OR = 0.68, 95% CI = 0.47 to 0.99). In a cochrane review [27] on condom use, 14 studies were included in the final analysis. The incidence of HIV infection among those who reported always using condoms was 1.14 per 100 person-years (95% CI= 0.56-2.04), while it was 5.75 per 100 person-years (95% CI= 3.16-9.66) among those who never used them. This gave an 80% reduction in the incidence of infection with condom use. It is however important to note that the meta-analysis was done using data from observational studies and the authors did not provide confidence intervals for their estimated effect of 80%.Observational studies inherently carry a risk of bias as Self-reported data always have the risk of being unreliable. Possible reasons for the absence of an association between HIV seroconversion with condom use in marriage, is that use of condoms in pregnancy is alien to our culture more so in pregnancy where contraception is unthinkable; and may lead to suspicion of infidelity. Cigarette smoking by a partner (AOR 3.0, 95% CI =0.95-9.4) was a significant predictor of HIV seroconversion in a rural hospital study in Northern Nigeria [23] following multivariate study, unlike in this study. Subjecting the study to Multivariate analysis, women of age group 25-29 years (OR: 3.20; 95%CI: 2.39-4.29) were most likely to seroconvert. Women married to men with sexually transmitted infections, as evidenced by penile discharge, were also associated with an increased risk of HIV seroconversion (OR: 09; 95%CI: 0.01-0.45; p =0.004). Same was the case of those in polygamous setting (OR: 3.98; CI: 1.64-9.65) in this study, which conforms to findings in the study in Northern Nigeria [23], South Africa [24] and Tanzania [22]. Polygamy remains a part of the African culture and Islam, as well as traditional religion. Polygamy is a ‘legalized’ way of having multiple sexual partners and so increases the risk of HIV transmission. Recreational drugs pose a risk for transmission of HIV especially in intravenous drug users who may share needles. It also affects the individual’s judgment thereby reducing a person's ability to make informed choices concerning safe sex and protection from HIV infection. There was an association in this study between recreational drug use and seroconversion; much similar to that found in a Mauritian study [28].

## Conclusion

The study revealed a seroconversion rate of 3% among booked, previously HIV negative clients, in labour. Eliminating mother to child transmission of HIV is still quite afar off. Predictors of sero-conversion in the study were patient’s age, alcohol intake, number of partner’s wives, partner’s use of recreational drugs and penile discharge in partners. Re-testing therefore becomes very necessary. Implementation of the recommendation concerning HIV retesting in labour as contained in the National Guidelines on PMTCT 2010, should be made top priority if elimination of MTCT is desired. All facilities in the country should be provided the wherewithal to implement this recommendation. A larger multicenter study involving urban and rural health facilities as well as the private health facilities, would give a clearer picture of the risk factors associated with HIV seroconversion.

### What is known about this topic

- HIV seroconversion can occur during pregnancy in a woman who was previously HIV negative;
- Many factors have been implicated in HIV seroconversion during the antenatal period.

### What this study adds

- The prevalence of HIV seroconversion among antenatal women in UUTH Uyo is significantly high to warrant routine HIV retesting in late pregnancy or labour;
- The common risk factors identified for HIV seroconversion among antenatal women in UUTH Uyo were patient’s age (25 -29 years), alcohol intake, number of partner’s wives, partner’s use of recreational drugs and penile discharge in partners;
- There is good knowledge (over 95%) of the mode of transmission of HIV among antenatal women in UUTH Uyo.
